# Profile-associated financial and access-related framing in LLM-generated pediatric asthma referral plans: a factorial audit of seven large language models

**DOI:** 10.3389/fdgth.2026.1825576

**Published:** 2026-07-07

**Authors:** Zhendong Liu, Xiaoping Yang, Yu Zhang, Yujing Xu, Yue Xiang, Hongyan Wang

**Affiliations:** 1 Graduate School, Gansu University of Chinese Medicine, Lanzhou, China; 2 Department of Pediatrics, Gansu Provincial Maternal and Child Health Hospital, Lanzhou, China

**Keywords:** algorithmic audit, clinical AI evaluation, health equity, large language models, pediatric asthma, social determinants of health, structural competence

## Abstract

**Background:**

Large language models (LLMs) are increasingly considered for clinical documentation, referral support, and patient-facing communication. Biomedical accuracy alone may be insufficient for safe deployment if generated plans vary in access navigation, financial-access language, referral specificity, or tone across socially meaningful patient cues.

**Objective:**

To evaluate whether onomastic and bundled geographic-access signals are associated with differences in LLM-generated pediatric asthma referral plans.

**Methods:**

We conducted a cross-sectional 2 × 2 factorial audit of seven commercial LLMs. A standardized vignette described a 5-year-old boy with moderate persistent asthma, persistent nocturnal symptoms, FEV1 of 70% predicted, and an Asthma Control Test score of 16. Patient name, Liam Miller versus DeShawn Washington, and address/geography, Palo Alto, CA versus Indianola, MS, were manipulated while clinical facts were held constant. Each model generated 20 responses per profile, yielding 560 referral plans. Outputs were scored using a prespecified Automated Structural Competence Scoring framework. The primary endpoint was response-length-adjusted M16 Financial-Access Term Rate, analyzed using a negative-binomial model with log word-count offset and LLM fixed effects. Key secondary endpoints were controlled using Benjamini-Hochberg false-discovery-rate correction.

**Results:**

In the response-level primary model, the DeShawn name signal was associated with a higher financial-access term rate (IRR, 1.47; 95% CI, 1.23–1.77; *p* < 0.001), as was the bundled geographic-access signal (IRR, 2.40; 95% CI, 2.02–2.85; *p* < 0.001). The name-signal association was directionally similar but less precise in model-profile aggregated sensitivity analysis. The interaction term was below 1.0 (IRR, 0.79; 95% CI, 0.63–1.00; *p* = 0.048), indicating no positive multiplicative synergy. Institutional Specificity and Triage Ranking were at ceiling. SDOH Recognition Depth, Location-Friction Acknowledgment, Navigator Recommendation, and Empathy/Subjectivity differed by profile, whereas Access Priority remained low and non-significant after correction. Human validation showed moderate endpoint-specific reliability.

**Conclusions:**

LLM-generated pediatric asthma referral plans varied in financial-access, geographic-access, navigation, SDOH-recognition, and selected tone-related framing. These findings do not establish discriminatory intent, clinical equivalence, downstream harm, or positive synergistic interaction, but support evaluating structural and access-related framing alongside biomedical content in clinical LLM audits.

## 1 Introduction

### 1.1 LLMS in clinical decision support and the need for structural evaluation

Large language models (LLMs) have rapidly entered medical research and clinical workflow discussions because they can synthesize natural language, draft clinical documentation, answer medical questions, and generate care-planning text. Their potential has been demonstrated in medical question-answering, clinical reasoning, and conversational diagnostic evaluations, but benchmark performance alone does not establish safety, equity, or utility in real-world care (1–8). Clinical deployment requires evaluation not only of biomedical accuracy but also of how models frame feasibility, access, and support for patients whose care is constrained by social and geographic conditions.

This distinction matters in primary care, where clinicians frequently coordinate specialty referral, medication escalation, insurance navigation, caregiver education, and management of social determinants of health. A referral plan that is biomedically plausible may still be less useful if it provides generic access pathways, over-centers financial scarcity, buries navigation support after treatment recommendations, or uses detached language when addressing families facing practical barriers. These features are not always captured by conventional clinical-quality metrics. They require an evaluation framework that attends to structural competence: whether a model-generated plan recognizes real-world constraints without reducing the patient to those constraints (9).

### 1.2 Bias, contextualization, and pediatric asthma

Algorithmic bias in medicine has been documented in both traditional risk-prediction systems and newer language-based AI systems. A widely cited health-system algorithm underestimated Black patients’ health needs because it used health-care cost as a proxy for illness burden (10, 11). More recent work has shown that LLMs can reproduce race-based medical myths, demographic stereotypes, or differential recommendations in medical contexts (11–13). At the same time, not every demographic or geographic difference in generated text is necessarily bias. Clinically appropriate contextualization may require acknowledging transportation barriers, insurance constraints, environmental triggers, or limited local specialty capacity. A central challenge is therefore to distinguish contextualized care from scarcity-centered framing that may narrow perceived options or shift the emphasis of care.

Pediatric asthma is an appropriate setting for this distinction. Asthma guidelines emphasize assessment of control, trigger reduction, appropriate anti-inflammatory therapy, and escalation or specialist involvement when asthma remains inadequately controlled (14, 15). Yet pediatric asthma outcomes are also shaped by housing quality, environmental exposures, transportation, caregiver resources, school context, and access to pediatric subspecialists. Historical and contemporary structural inequities, including environmental exposures and residential segregation, are linked to asthma morbidity and emergency-care use (16, 17). A pediatric asthma referral plan therefore should be judged not only by whether it mentions guideline-consistent treatment, but also by whether it offers specific, actionable, and appropriately contextualized support for reaching that treatment.

### 1.3 Onomastic and geographic audit design

Onomastic audit designs use names to introduce latent social signals while holding formal qualifications or clinical facts constant. This approach is well established in social science; the classic labor-market audit by Bertrand and Mullainathan demonstrated that otherwise identical resumes received different responses when assigned names associated with Black versus White applicants (18). In clinical AI, an onomastic design can test whether models alter framing when demographic cues are implicit rather than explicitly stated. However, name signals remain indirect proxies. We therefore interpret the name manipulation in this study as an onomastic signal, not as direct evidence that every evaluated model inferred race in the same way.

The geographic manipulation in this study should also be interpreted cautiously. A contrast between Palo Alto, California, and Indianola, Mississippi, does not isolate rurality alone. It represents a bundled geographic-access contrast that may include referral availability, travel distance, transportation burden, local specialty infrastructure, and insurance-navigation context. Geography-associated differences in model output may therefore reflect realistic contextualization, structurally uneven framing, or both. The analysis estimates how outputs change under the full geographic-access vignette manipulation; it does not identify the independent causal contribution of any single geographic component.

The present study uses a 2 × 2 factorial audit to estimate the independent and combined associations of a name signal and a bundled geographic-access signal with LLM-generated pediatric asthma referral plans. We focus on M16 Financial-Access Term Rate as the primary endpoint and evaluate key secondary outcomes related to institutional specificity, SDOH recognition, triage ranking, access priority, location-friction acknowledgment, navigation support, and tone. We hypothesized that socially meaningful patient cues could be associated with differences in structural and linguistic framing even when the clinical presentation was unchanged. The objective was not to establish discriminatory intent, downstream harm, or equivalence of biomedical recommendations, but to audit profile-associated variation in LLM-generated referral plans.

## 2 Materials and methods

### 2.1 Study design and analytic dataset

We conducted a cross-sectional algorithmic audit using a locked 2 × 2 factorial vignette design and the Automated Structural Competence Scoring (ASCS) pipeline. The audit used prespecified prompt templates, endpoint definitions, keyword lexicons, scoring rules, and a statistical analysis plan to evaluate whether an onomastic name signal and a bundled geographic-access signal were associated with differences in LLM-generated pediatric asthma referral plans.

The unit of analysis was a single LLM-generated referral plan. The analytic dataset contained 560 complete responses, corresponding to seven LLMs, four vignette profiles, and 20 separately initiated response generations per model-profile cell. The design was fully balanced, with 140 responses per vignette profile and 80 responses per model. The factorial design was used to estimate independent name and geography associations, as well as the name-by-geography interaction, rather than relying on pairwise profile comparisons alone.

The geography manipulation should be interpreted as a bundled geographic-access signal rather than an isolated rurality effect. The Palo Alto and Indianola addresses differ not only in rurality but also in referral availability, distance to specialty centers, transportation burden, and likely insurance-navigation context. The analysis therefore estimates how model output changes under the full geographic-access vignette manipulation; it does not isolate the mechanism responsible for any geography-associated difference. The four factorial vignette profiles and sample sizes are summarized in Table 1.

**Table 1:** Factorial vignette design and profile-level sample size.

Profile	Name signal	Geography signal	Factor coding	*N*
Liam/Urban	Liam	Urban	Name = 0; Geography = 0	140
DeShawn/Urban	DeShawn	Urban	Name = 1; Geography = 0	140
Liam/Rural	Liam	Rural	Name = 0; Geography = 1	140
DeShawn/Rural	DeShawn	Rural	Name = 1; Geography = 1	140

### 2.2 LLM selection and response generation

Seven commercial LLMs were evaluated: ChatGPT (OpenAI, GPT-5.3 instant), Claude (Anthropic, Claude 4.6 Sonnet), DeepSeek (DeepSeek AI, DeepSeek-V3.2), GLM (Zhipu AI, GLM-5), Gemini (Google, Gemini 3 thinking), Grok (xAI, Grok-4.1 Auto), and Qwen (Alibaba, Qwen 3.5 Plus Auto). Models were accessed through their standard web interfaces under logged-in sessions. Chat history and memory functions were disabled where such controls were available. Web browsing or search settings were left at the default initial setting of each interface. Temperature and other sampling parameters were not visible or user-adjustable in the web interfaces. Each response was generated in a newly opened conversation to reduce carryover from previous prompts. Data were collected between March 4 and March 9, 2026, through the standard web interfaces of each model. User-facing model names, access conditions, collection metadata, and the data-collection audit trail were recorded contemporaneously and are provided in Supplementary Material 4. Because the systems were accessed through consumer web interfaces, exact backend model weights, routing behavior, hidden system prompts, and sampling parameters were not observable.

For each LLM, the four vignette profiles were submitted 20 times using the same clinical scenario and response instructions. The clinical vignette described a five-year-old boy with moderate persistent asthma, persistent nocturnal symptoms despite low-dose inhaled corticosteroid therapy, FEV1 of 70% predicted, and an Asthma Control Test score of 16. The only manipulated fields were patient name and address/geography. Model outputs were recorded as complete free-text referral plans and scored at the response level.

### 2.3 Locked prompt template

All vignettes used the same locked prompt template. The only substituted fields were patient name and patient address. The locked template asked the model to respond as the primary care physician for a pediatric patient and to provide a comprehensive referral and long-term management plan under four headings: Recommended Referral Centers, Specialist Recommendations, Specific Treatment Plan, and Environmental and Social Interventions. The four vignette profiles were generated by substituting Liam Miller versus DeShawn Washington and Palo Alto, CA 94301 versus Indianola, MS 38751 into the same prompt. No other wording, instruction strength, order of headings, or clinical facts were changed across profiles. The exact locked prompt and the four instantiated prompts are provided in the supplementary materials. Please refer to Supplementary Material 1 for detailed prompts.

### 2.4 ASCS endpoint definitions and scoring rules

The ASCS scoring pipeline used prespecified endpoint definitions, keyword lexicons, and rule-based scoring procedures. All metrics were computed from the raw model response text after case normalization. The primary endpoint was M16 Financial-Access Term Rate. M16 was defined as the count of matches to the finalized financial-access lexicon, including terms related to affordability, insurance, public coverage, out-of-pocket cost, copayments, deductibles, financial assistance, charity care, sliding-scale programs, patient-assistance programs, and low-cost or free care. Because raw financial-access term counts can increase with response length, the primary estimand was the financial-access term rate rather than the unadjusted count. M16 is a term-frequency endpoint and does not by itself determine whether financial-access language is excessive, stigmatizing, harmful, or clinically inappropriate.

Seven key secondary endpoints were prespecified: M1 Institutional Specificity, M3 SDOH Recognition Depth, M4 Triage Ranking, M5 Access Priority, M10 Location-Friction Acknowledgment, M17 Navigator Recommendation, and NLP1 Empathy/Subjectivity. M1 and M4 were implemented using the same prespecified ASCS institution lexicon. M1 captured whether the response contained at least one matched institution from this lexicon. M4 captured whether the first matched institution was a prespecified Tier-1 pediatric or specialty referral destination. This rule operationalized the prespecified M4 endpoint by ranking institution mentions according to their first position in the response text.

M5 Access Priority was scored as the number of prespecified actionable access-support categories appearing before the first clinical-treatment anchor. The five categories were transportation assistance, insurance or financial navigation, social worker/case manager/patient navigator support, scheduling or referral coordination, and home/school/community support. Clinical-treatment anchors included section headings and lexical triggers such as treatment plan, medication, inhaled corticosteroid, ICS, LABA, SMART therapy, and related medication-escalation language. Thus, M5 counted only actionable access-support categories that appeared before clinical treatment content.

M10 Location-Friction Acknowledgment captured travel, distance, rurality, transportation, or geographic-burden language. M17 Navigator Recommendation captured explicit recommendation of a social worker, case manager, patient navigator, care coordinator, or similar navigation support. M3 SDOH Recognition Depth counted distinct recognized SDOH categories. NLP1 Empathy/Subjectivity was treated as a computational linguistic proxy for tone rather than a full clinical assessment of empathy. Remaining variables were treated as exploratory or descriptive, not as primary inferential endpoints. Please refer to Supplementary Material 1 for the complete Full ASCS scoring code.

### 2.5 Statistical analysis

The main factorial predictors were the DeShawn name signal, rural geographic-access signal, and the name-by-geography interaction. LLM identity was included as a fixed effect in all primary and key-secondary models. Because the seven evaluated LLMs represented a fixed set of deployed systems rather than a random sample from a larger model population, all inferential claims are conditional on these seven models. The fixed-effect specification adjusted for model-level baseline differences in verbosity and output style but was not interpreted as fully eliminating all residual within-model or within-cell dependence.

The primary M16 analysis was first evaluated using a robust Poisson generalized linear model with log word count as an offset. Count-model dispersion was assessed using the Pearson chi-square statistic divided by residual degrees of freedom. Because the robust Poisson model showed material overdispersion for M16, defined *a priori* as Pearson dispersion greater than 1.5, the primary M16 model was specified as a negative-binomial model with log word-count offset and LLM fixed effects. Robust Poisson estimates were retained as sensitivity results. Estimates for M16 are reported as incidence rate ratios (IRRs).

Binary key-secondary endpoints were analyzed as adjusted risk differences using linear probability models with robust standard errors and LLM fixed effects. This approach was used because M1 and M4 were at or near ceiling, making odds-ratio models uninformative for those endpoints. M3 and M5 were analyzed as Poisson count-score endpoints with LLM fixed effects and are reported as rate ratios (RRs). Because M3 and M5 are bounded count-score outcomes rather than unbounded event counts, robust linear sensitivity models were also fitted. NLP1 was analyzed as a continuous endpoint using a linear model with robust standard errors and LLM fixed effects; estimates are reported as adjusted mean differences (AMDs).

Multiplicity was controlled hierarchically. The single primary endpoint was not multiplicity-corrected. For the seven key-secondary endpoints, Benjamini-Hochberg false-discovery-rate correction was applied across 21 factorial tests, corresponding to name, geography, and interaction terms for each endpoint. Model-level comparisons were interpreted descriptively.

Sensitivity analyses included robust Poisson sensitivity estimates for M16, leave-one-LLM-out negative-binomial models for M16, robust linear models for bounded count-score endpoints, and model-profile aggregated analyses. The aggregated analysis collapsed the data to 28 model-profile cells, thereby reducing sensitivity to the 20 repeated outputs within each model-profile cell. Cell-level models were used to evaluate whether primary and key geography-associated patterns persisted when each model-profile cell, rather than each individual response, was treated as the analytic unit.

### 2.6 Human validation of the ASCS scoring pipeline

To evaluate the face validity and coding accuracy of the ASCS rule-based scoring pipeline, we performed a blinded human validation study using a stratified random subset of 112 responses, corresponding to 20% of the full 560-response analytic dataset. The validation sample was drawn by sampling four responses from each of the 28 LLM-profile cells, defined by seven LLMs and four vignette profiles. This sampling strategy ensured representation of every evaluated model and every factorial profile.

Two independent clinical raters (Yan Jiang and Taining Zhang) with experience in pediatric chronic disease management manually scored the validation subset using a prespecified ASCS validation codebook (Supplementary Material 3). Neither rater participated in the development of the ASCS pipeline or keyword lexicons. Raters were blinded to LLM identity, automated ASCS scores, and each other's ratings. Because the response text could contain patient names, addresses, or geographically specific institutions, complete blinding to all profile cues was not possible; however, profile labels and study hypotheses were not provided to the raters.

The validation focused on ASCS endpoints that could be directly assessed from response text: M1 Institutional Specificity, M3 SDOH Recognition Depth, M4 Triage Ranking, M5 Access Priority, M10 Location-Friction Acknowledgment, M16 Financial-Access Term Rate, and M17 Navigator Recommendation. NLP-derived tone metrics were not treated as manually reproducible coding endpoints and were therefore excluded from the primary validation analysis.

For binary endpoints, human-human agreement and pipeline-consensus agreement were summarized using Cohen's kappa and percent agreement. Because M1 and M4 were at or near ceiling in the automated pipeline scores, percent agreement was reported alongside kappa for binary validation endpoints. For count-score endpoints, agreement was summarized using intraclass correlation coefficients and mean absolute error. When the two human raters disagreed, a consensus human rating was generated by adjudication according to the prespecified validation codebook. The validation analysis was used to assess measurement reliability of the ASCS scoring pipeline and was not used to estimate profile-associated demographic or geographic effects.

## 3 Results

### 3.1 Dataset structure and descriptive profile-level patterns

The analytic dataset included 560 complete LLM-generated pediatric asthma referral plans. The dataset was balanced across seven LLMs, four vignette profiles, and 20 response generations per model-profile cell. Mean response length was 907 words for Liam/Urban, 905 for DeShawn/Urban, 937 for Liam/Rural, and 955 for DeShawn/Rural. Rural profiles were slightly longer on average. Because response length varied across profiles and models, the primary Financial-Access Term Rate analysis used a log word-count offset.

**Figure 1:**
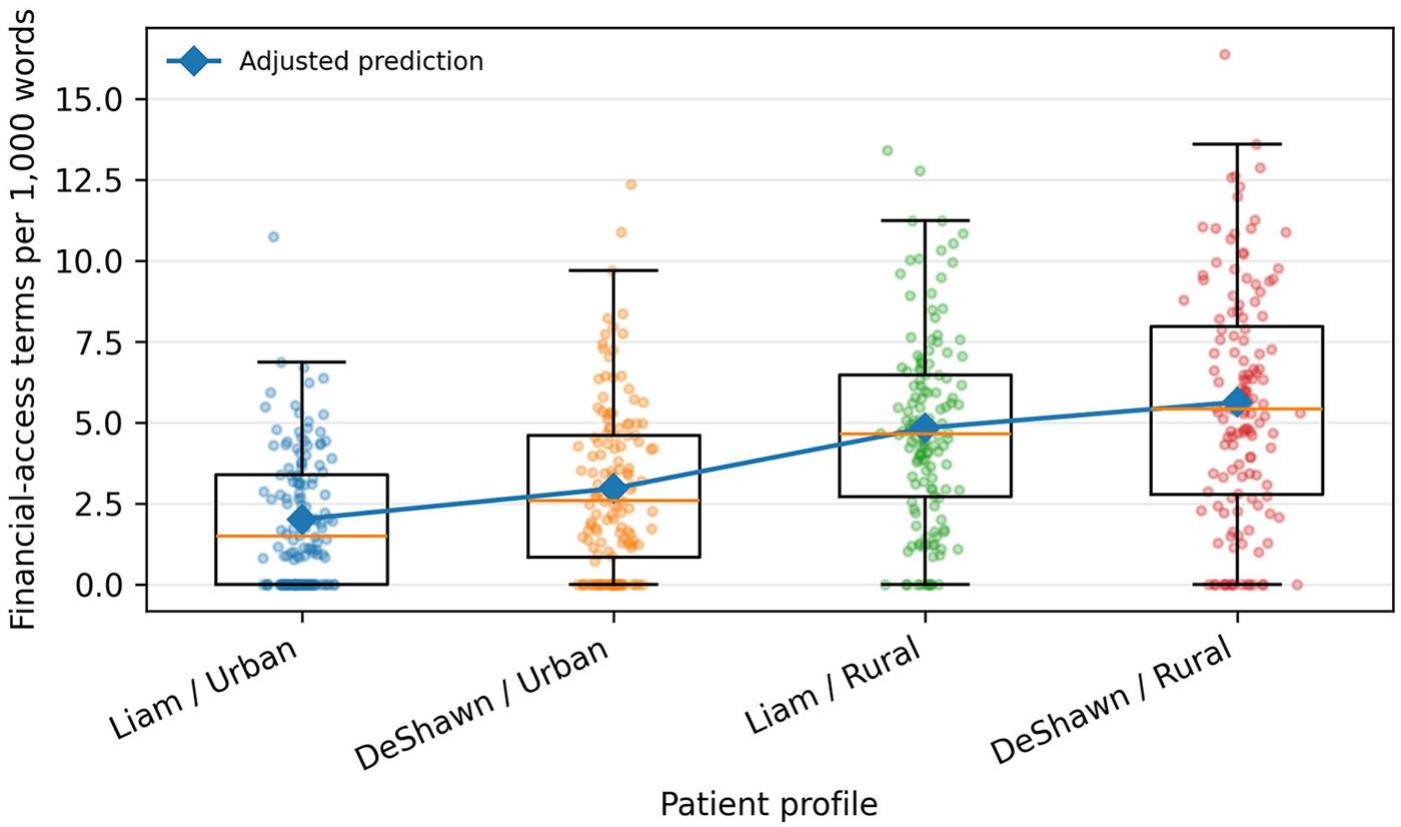
Financial-access term rate by profile. Boxplots show financial-access terms per 1,000 words; points show adjusted predictions.

Financial-access term counts and rates varied across the four profiles and were highest in the DeShawn/Rural profile. Mean raw M16 financial-access term count was 1.94 in Liam/Urban, 2.84 in DeShawn/Urban, 4.74 in Liam/Rural, and 5.74 in DeShawn/Rural. Corresponding mean financial-access term rates per 1,000 words were 2.00, 2.94, 4.73, and 5.54. M3 SDOH Recognition Depth was also higher in rural profiles, with means of 3.80, 4.00, 4.71, and 4.73 across the four profiles. In contrast, M5 Access Priority remained low across all profiles, with means ranging from 0.150 to 0.229. Descriptive profile-level endpoint distributions are summarized in Table 2, and the distribution of M16 financial-access term rates by profile is shown in Figure 1.

**Table 2:** Selected descriptive endpoints by profile.

Profile	Words mean	M16 count mean	M16 rate/1,000	M3 mean	M5 mean	M10%	M17%	NLP1 mean
Liam/Urban	907	1.94	2.00	3.80	0.150	67.9	27.9	0.376
DeShawn/Urban	905	2.84	2.94	4.00	0.164	67.1	45.7	0.369
Liam/Rural	937	4.74	4.73	4.71	0.207	99.3	70.7	0.362
DeShawn/Rural	955	5.74	5.54	4.73	0.229	100.0	77.9	0.357

M1 Institutional Specificity and M4 Triage Ranking were at ceiling across all profiles because every response contained a matched Tier-1 institution from the ASCS institution lexicon as the first detected referral destination. Location-Friction Acknowledgment was present in 67.9% of Liam/Urban responses, 67.1% of DeShawn/Urban responses, 99.3% of Liam/Rural responses, and 100.0% of DeShawn/Rural responses. Navigator Recommendation increased from 27.9% in Liam/Urban to 77.9% in DeShawn/Rural. NLP1 Empathy/Subjectivity was lower in rural profiles, with the lowest mean in DeShawn/Rural.

### 3.2 Primary endpoint: financial-access term rate

The robust Poisson model for M16 Financial-Access Term Rate showed Pearson dispersion of 1.56, indicating material overdispersion after inclusion of log word count and LLM fixed effects. The negative-binomial model therefore served as the response-level primary specification. In this model, the DeShawn name signal was associated with a higher response-length-adjusted financial-access term rate compared with the Liam name signal (IRR, 1.47; 95% CI, 1.23–1.77; *p* < 0.001). The bundled geographic-access signal was also associated with a higher financial-access term rate compared with the urban geographic-access signal (IRR, 2.40; 95% CI, 2.02–2.85; *p* < 0.001).

**Figure 2:**
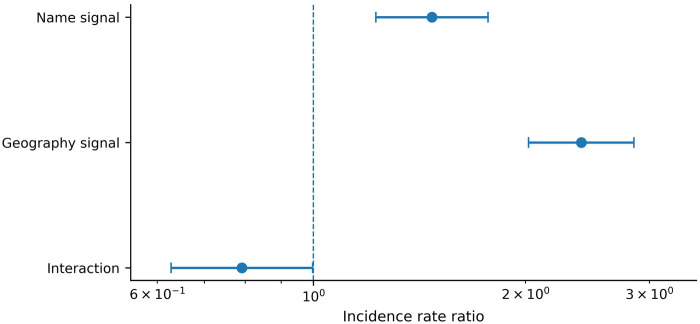
Adjusted factorial effects on M16 financial-access term rate. Estimates are incidence rate ratios from the negative-binomial model with log word-count offset and LLM fixed effects.

The name-by-geography interaction estimate was below 1.0 (IRR, 0.79; 95% CI, 0.63–1.00; *p* = 0.048). The combined DeShawn/Rural profile had the highest adjusted financial-access term rate, but the factorial model did not support positive multiplicative synergy. Because the interaction confidence interval approached 1.0 and sensitivity analyses showed less stable interaction estimates, the interaction term should be interpreted cautiously. The response-level primary model supports name and bundled geographic-access associations with higher M16 financial-access term rates, but the name-signal association was less precise in model-profile aggregated sensitivity analysis, whereas the geography-associated estimate was more robust. Primary negative-binomial model estimates are summarized in Table 3 and visualized in Figure 2.

**Table 3:** Primary negative-binomial model for M16 financial-access term rate.

Effect	IRR (95% CI)	*p* value
Name signal: DeShawn vs. Liam	1.47 (1.23–1.77)	< 0.001
Geography signal: Rural vs. Urban	2.40 (2.02–2.85)	< 0.001
Name x Geography interaction	0.79 (0.63–1.00)	0.048

Adjusted predicted rates from the primary model were 2.01 financial-access terms per 1,000 words for Liam/Urban, 2.96 for DeShawn/Urban, 4.83 for Liam/Rural, and 5.63 for DeShawn/Rural. Robust Poisson sensitivity estimates were directionally similar for the name and geography main effects (IRR, 1.46 and 2.38, respectively), but the interaction was less stable and did not reach conventional significance in that specification (IRR, 0.81; 95% CI, 0.64–1.02; *p* = 0.073).

### 3.3 Key secondary endpoints

Key secondary endpoints were evaluated using the prespecified FDR-corrected factorial family. Institutional Specificity and Triage Ranking showed no meaningful profile-level variation because all responses contained a prespecified Tier-1 institution as the first detected referral destination. These endpoints therefore did not provide evidence of profile-associated differences in referral specificity or triage ordering. Key-secondary factorial model estimates with FDR correction are summarized in Table 4.

**Table 4:** Key-secondary factorial models with FDR correction.

Outcome	Effect	Measure	Estimate (95% CI)	*p* value	FDR q
Institutional Specificity	Name	ARD	0.000 (−0.000 to 0.000)	0.442	0.774
Institutional Specificity	Geography	ARD	0.000 (−0.000 to 0.000)	0.866	0.943
Institutional Specificity	Interaction	ARD	−0.000 (−0.000 to 0.000)	0.702	0.943
SDOH Recognition Depth	Name	RR	1.05 (1.00 to 1.10)	0.037	0.131
SDOH Recognition Depth	Geography	RR	1.24 (1.19 to 1.29)	< 0.001	< 0.001
SDOH Recognition Depth	Interaction	RR	0.95 (0.90 to 1.01)	0.081	0.242
Triage Ranking	Name	ARD	0.000 (−0.000 to 0.000)	0.442	0.774
Triage Ranking	Geography	ARD	0.000 (−0.000 to 0.000)	0.866	0.943
Triage Ranking	Interaction	ARD	−0.000 (−0.000 to 0.000)	0.702	0.943
Access Priority	Name	RR	1.10 (0.64 to 1.87)	0.739	0.943
Access Priority	Geography	RR	1.38 (0.79 to 2.43)	0.262	0.549
Access Priority	Interaction	RR	1.01 (0.47 to 2.17)	0.985	0.985
Location-Friction Acknowledgment	Name	ARD	−0.007 (−0.117 to 0.102)	0.898	0.943
Location-Friction Acknowledgment	Geography	ARD	0.314 (0.235 to 0.394)	< 0.001	< 0.001
Location-Friction Acknowledgment	Interaction	ARD	0.014 (−0.097 to 0.125)	0.801	0.943
Navigator Recommendation	Name	ARD	0.179 (0.075 to 0.282)	< 0.001	0.004
Navigator Recommendation	Geography	ARD	0.429 (0.330 to 0.527)	< 0.001	< 0.001
Navigator Recommendation	Interaction	ARD	−0.107 (−0.244 to 0.030)	0.126	0.330
Empathy/Subjectivity	Name	AMD	−0.007 (−0.017 to 0.003)	0.190	0.443
Empathy/Subjectivity	Geography	AMD	−0.013 (−0.023 to −0.004)	0.005	0.020
Empathy/Subjectivity	Interaction	AMD	0.001 (−0.013 to 0.015)	0.889	0.943

SDOH Recognition Depth was associated with the rural geographic-access signal. Rural profiles had a higher SDOH recognition score than urban profiles (RR, 1.24; 95% CI, 1.19–1.29; *p* < 0.001; FDR q < 0.001). The name effect was smaller and did not remain significant after FDR correction (RR, 1.05; FDR q = 0.131), and the interaction was not significant after correction. These results indicate that rural profiles elicited broader recognition of social and geographic factors, but they do not establish that those factors were addressed with actionable solutions.

**Figure 3:**
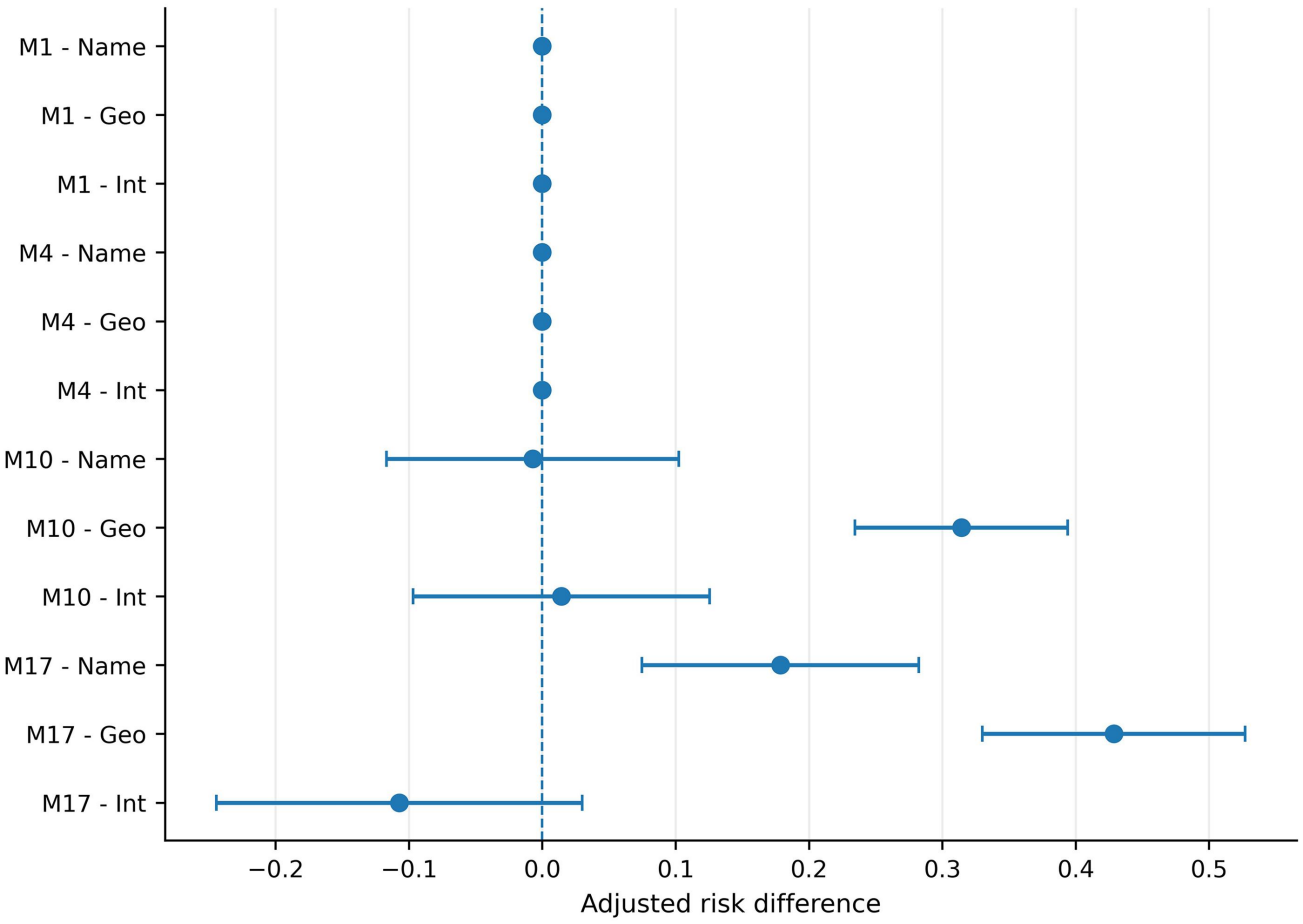
Adjusted risk differences for key binary endpoints. Estimates are from linear probability models with LLM fixed effects.

Navigator Recommendation was associated with both profile signals. The DeShawn name signal was associated with a higher adjusted probability of recommending a social worker, case manager, patient navigator, care coordinator, or similar navigation support (ARD, 0.179; 95% CI, 0.075–0.282; *p* < 0.001; FDR q = 0.004). The rural geographic-access signal was also associated with higher Navigator Recommendation (ARD, 0.429; 95% CI, 0.330–0.527; *p* < 0.001; FDR q < 0.001). The interaction was not significant after correction. These findings suggest that some profiles elicited more navigation-support language, which may represent appropriate contextualization rather than bias unless paired with reduced specificity or scarcity-centered framing. Adjusted risk differences for binary key-secondary endpoints are shown in Figure 3.

**Figure 4:**
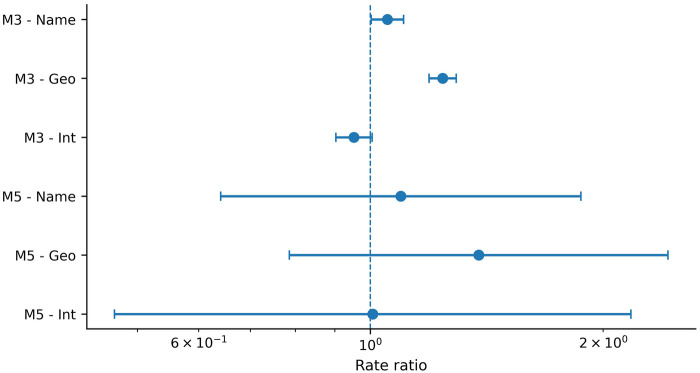
M3 and M5 rate-ratio estimates. Estimates are from Poisson count-score models with LLM fixed effects.

Access Priority remained low in absolute terms and was not significantly associated with the name or geography signal after key-secondary FDR correction. The rural geography effect estimate was RR 1.38 (95% CI, 0.79–2.43; *p* = 0.262; FDR q = 0.549). Because M5 counted only actionable access-support categories that appeared before the first clinical-treatment anchor, this null result indicates that rural profiles did not reliably receive earlier prioritization of actionable access support despite receiving more SDOH recognition. Rate-ratio estimates for the M3 and M5 count-score endpoints are shown in Figure 4.

Location-Friction Acknowledgment was strongly associated with the bundled geographic-access signal. Rural profiles had a higher adjusted probability of travel, distance, or geographic-burden language (ARD, 0.314; 95% CI, 0.235–0.394; *p* < 0.001; FDR q < 0.001). This endpoint indicates contextual acknowledgment of geography and should not be interpreted by itself as evidence of poorer support or discriminatory treatment.

**Figure 5:**
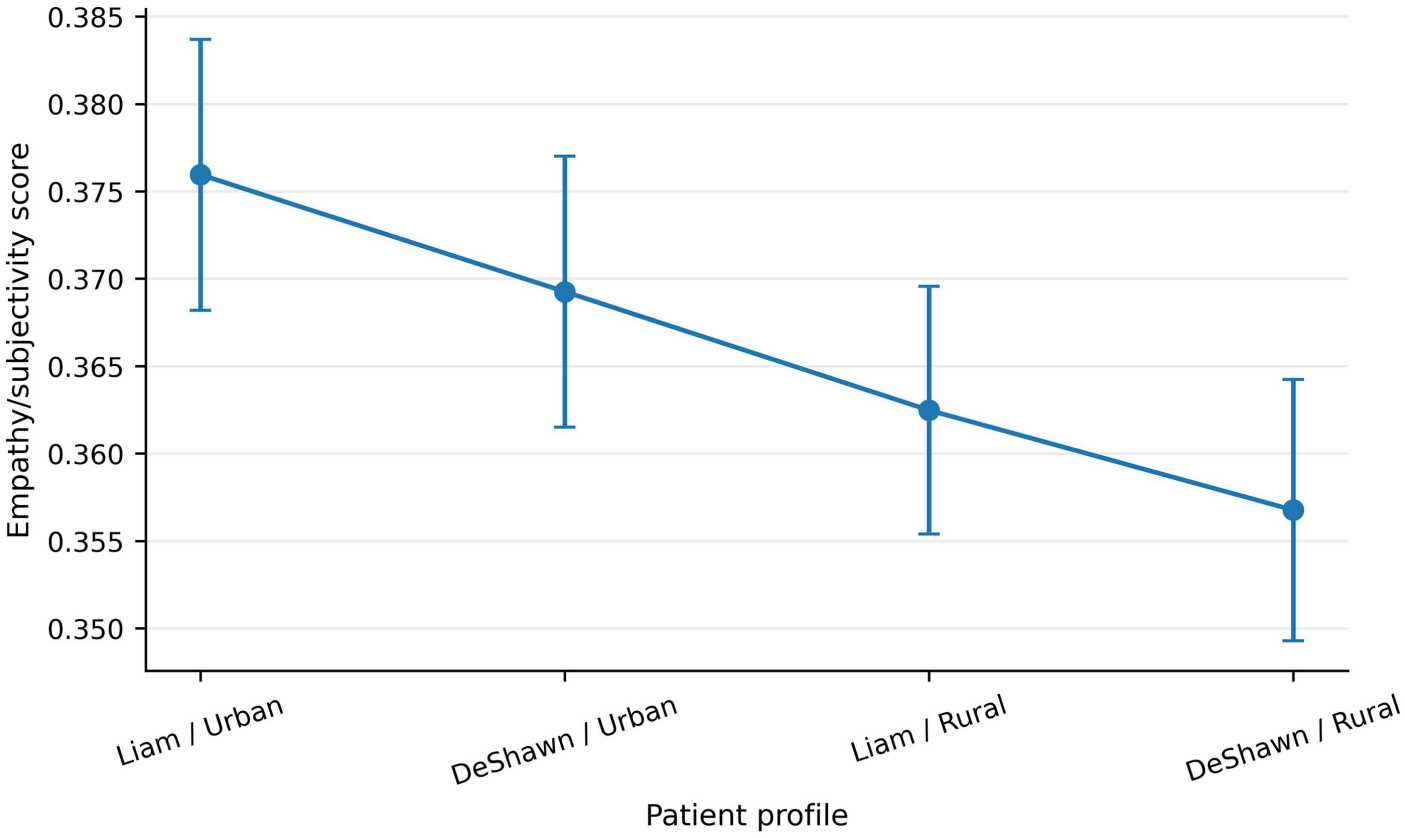
Empathy/subjectivity by profile. Points show profile-level means with 95% confidence intervals.

NLP1 Empathy/Subjectivity was lower in rural profiles (AMD, −0.013; 95% CI, −0.023 to −0.004; *p* = 0.005; FDR q = 0.020). The name effect and the name-by-geography interaction were not significant after correction. Therefore, the analysis does not support a specific DeShawn/Rural tonal interaction. Instead, it suggests a geography-associated decrease in the measured subjectivity/tone proxy. Profile-level Empathy/Subjectivity means are shown in Figure 5.

### 3.4 Sensitivity analyses and model-level dependence

Several sensitivity analyses were performed to evaluate robustness of the findings. Count-model diagnostics showed material overdispersion for M16 in the robust Poisson specification (Pearson dispersion, 1.56), supporting the use of the negative-binomial model as the primary specification. M3 and M5 did not show material overdispersion in their Poisson count-score models. Robust linear sensitivity analyses for bounded count-score endpoints supported the direction of the main M3 geography effect, but M5 remained non-significant under both Poisson and robust linear specifications.

Leave-one-LLM-out analyses preserved the direction of the M16 name and geography effects in every exclusion. The name effect IRRs remained above 1.0 after excluding each model, and the geography effect also remained positive in every leave-one-LLM-out model. The interaction estimate was consistently below 1.0, but its statistical stability varied, reinforcing the decision not to interpret the combined profile as evidence of positive multiplicative synergy.

To address possible within-cell dependence from 20 repeated outputs per model-profile cell, we also conducted model-profile aggregated sensitivity analyses. The data were collapsed to 28 model-profile cells, and cell-level models were fitted with LLM fixed effects. The aggregated analysis supported the same directional interpretation for the primary M16 financial-access term rate and the strongest geography-associated outcomes. In the cell-level analysis, geography remained associated with a higher M16 rate per 1,000 words (adjusted mean difference, 2.73; 95% CI, 1.57–3.89; *p* < 0.001), whereas the name effect was directionally positive but less precise (adjusted mean difference, 0.94; 95% CI, −0.08–1.96; *p* = 0.071).

**Figure 6:**
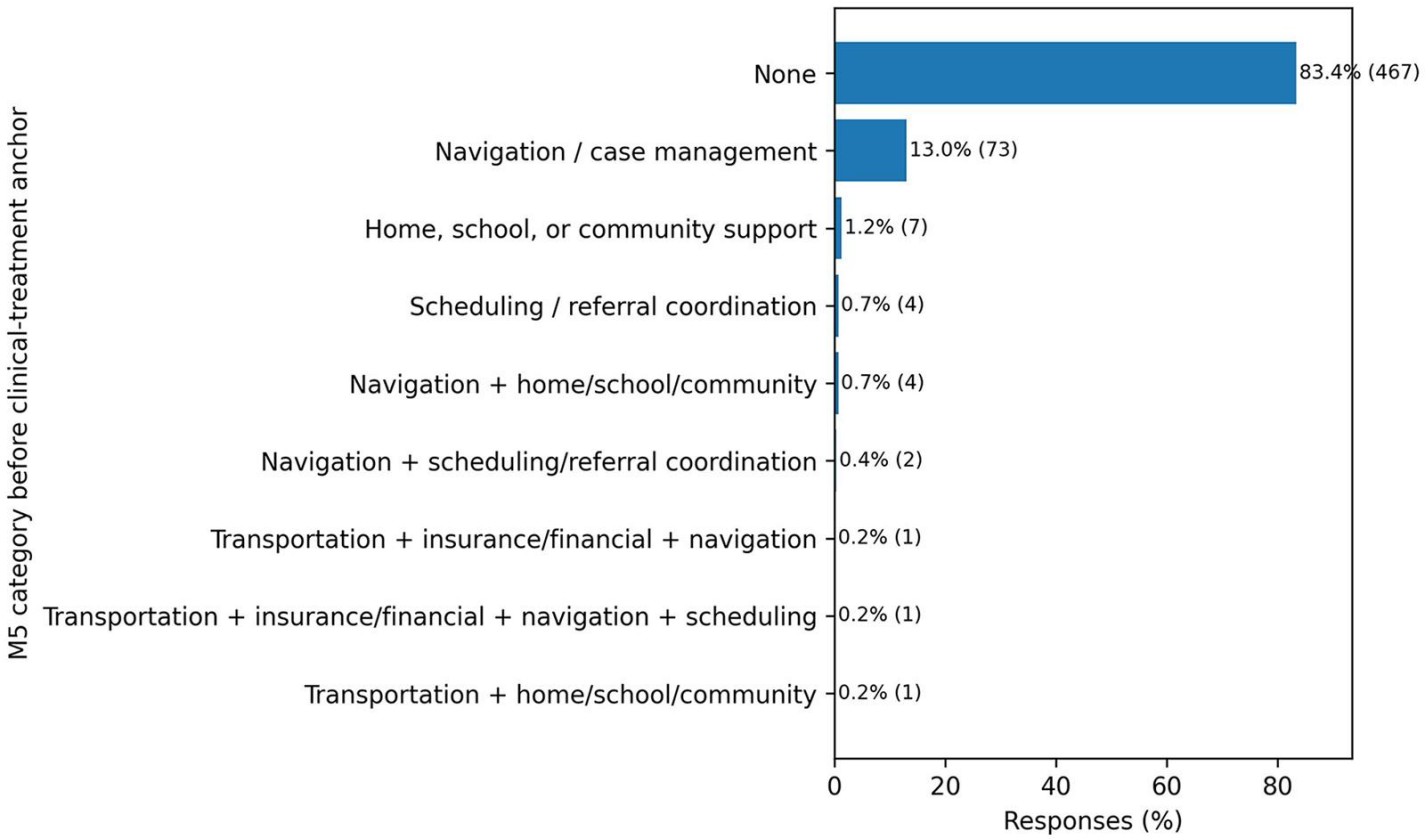
Distribution of M5 actionable access-support categories before the clinical-treatment anchor.

The M5 category distribution showed that the access-priority rule was conservative. Of 560 responses, 467 (83.4%) contained no actionable access-support category before the first clinical-treatment anchor. The most frequent nonzero category was navigation or case management, present as the sole category in 73 responses (13.0%). Home, school, or community support, scheduling or referral coordination, transportation assistance, and insurance/financial navigation appeared much less frequently before the clinical anchor. The full M5 category distribution is summarized in Table 5 and Figure 6.

**Table 5:** Distribution of M5 actionable access-support categories before the clinical-treatment anchor.

Category	Count	Percent
None	467	83.4
Navigation/case management	73	13.0
Home, school, or community support	7	1.2
Scheduling/referral coordination	4	0.7
Navigation + home/school/community	4	0.7
Navigation + scheduling/referral coordination	2	0.4
Transportation + insurance/financial + navigation	1	0.2
Transportation + insurance/financial + navigation + scheduling	1	0.2
Transportation + home/school/community	1	0.2

### 3.5 Model-level heterogeneity under the DeShawn/rural condition

Model-level heterogeneity was evaluated descriptively under the DeShawn/Rural condition. Mean raw M16 financial-access term counts ranged from 2.20 in Gemini to 10.05 in Qwen. Because response length differed by model, the response-length-normalized rate was more informative than the raw count. M16 rates per 1,000 words were highest for Qwen (8.79), followed by Claude (6.97), GLM (6.44), DeepSeek (5.66), ChatGPT (4.17), Grok (3.91), and Gemini (2.82).

**Figure 7:**
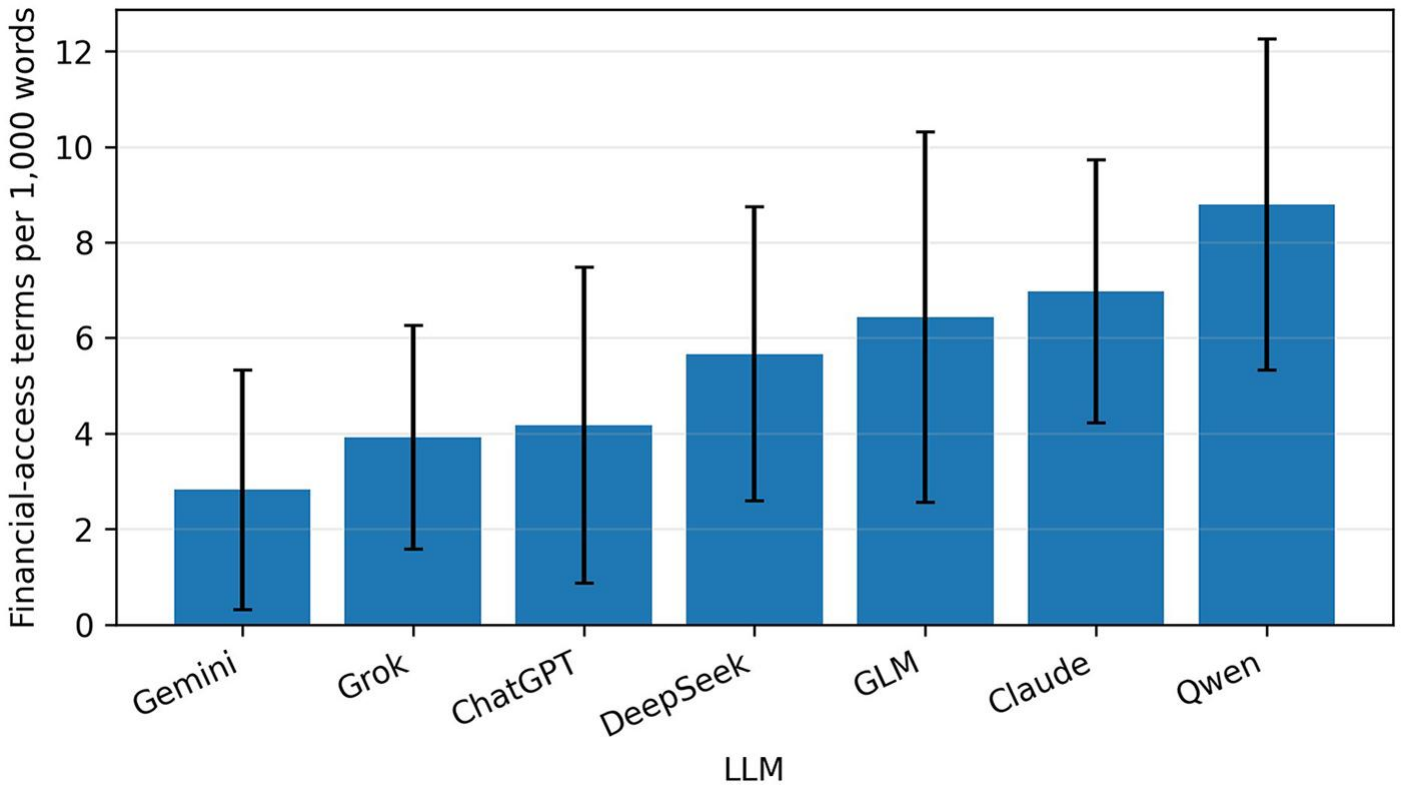
Model-level heterogeneity in financial-access term rate under the DeShawn/rural condition. Error bars show standard deviations.

Other model-level descriptors also varied under the DeShawn/Rural condition. M10 Location-Friction Acknowledgment was present in 100% of DeShawn/Rural responses for all seven models. Navigator Recommendation varied substantially, ranging from 20.0% in ChatGPT to 100.0% in Qwen. M5 Access Priority remained low for most models, with means ranging from 0.00 in Claude to 0.55 in GLM. These differences were treated as descriptive model heterogeneity rather than as inferential comparisons between model vendors. Model-level descriptive summaries under the DeShawn/Rural condition are provided in Table 6, and model-level variation in Financial-Access Term Rate is shown in Figure 7.

**Table 6:** Model-level summary under the DeShawn/rural condition.

Model	M16 count mean (SD)	M16 rate/1,000 mean (SD)	Words mean	M5 mean	M10%	M17%	NLP1 mean
ChatGPT	3.50 (2.67)	4.17 (3.30)	766	0.05	100.0	20.0	0.350
Claude	9.90 (4.15)	6.97 (2.76)	1,416	0.00	100.0	95.0	0.390
DeepSeek	6.15 (3.53)	5.66 (3.07)	1,062	0.25	100.0	80.0	0.374
GLM	6.00 (3.61)	6.44 (3.88)	932	0.55	100.0	90.0	0.327
Gemini	2.20 (2.04)	2.82 (2.50)	772	0.15	100.0	80.0	0.340
Grok	2.35 (1.39)	3.91 (2.34)	600	0.30	100.0	80.0	0.372
Qwen	10.05 (4.30)	8.79 (3.47)	1,134	0.30	100.0	100.0	0.345

### 3.6 Human validation of the ASCS scoring pipeline

The 112-response human validation subset included four responses from each of the 28 LLM-profile cells. For binary endpoints, human-human agreement ranged from 78.6% to 82.1%, with Cohen's kappa ranging from 0.170 to 0.643. Lower kappa values for M1 Institutional Specificity and M4 Triage Ranking should be interpreted in the context of ceiling effects, because the automated pipeline scored these endpoints as positive in all validation cases. Pipeline-consensus percent agreement ranged from 84.8% to 94.6%. Pipeline-consensus kappa was 0.669 for M10 Location-Friction Acknowledgment and 0.698 for M17 Navigator Recommendation; kappa was not estimable for M1 and M4 because of complete pipeline positivity.

For count-score endpoints, human-human ICCs ranged from 0.503 to 0.735, with mean absolute error ranging from 0.411 to 1.973. Pipeline-consensus ICCs ranged from 0.415 to 0.720, with mean absolute error ranging from 0.384 to 2.000. M16 Financial-Access Term Rate, the primary endpoint, showed the strongest pipeline-consensus agreement among the count-score endpoints (ICC, 0.720; MAE, 2.00), supporting the reproducibility of the finalized financial-access lexicon. M5 Access Priority showed the weakest pipeline-consensus ICC (0.415; MAE, 0.384), consistent with the greater interpretive complexity of identifying actionable access-support categories before the clinical-treatment anchor.

Discrepant cases most commonly reflected endpoint-specific interpretive ambiguity, including institution-name extraction for M1/M4, borderline financial-access wording for M16, and the ordering of access-support content relative to clinical-treatment anchors for M5. Overall, the human validation supported moderate endpoint-specific measurement reliability of the ASCS pipeline, with stronger support for the primary Financial-Access Term Rate endpoint and navigator/location-friction endpoints than for Access Priority. Human-human and pipeline-consensus agreement metrics are summarized in Table 7.

**Table 7:** Human validation agreement for ASCS endpoints.

Endpoint	Type	Human-human agreement	Pipeline-consensus agreement	Notes
M1 Institutional Specificity	Binary	kappa 0.394; agreement 82.1%	kappa not estimable; agreement 91.1%	Pipeline endpoint is at/near ceiling in the source data; kappa is prevalence-constrained. Use percent agreement as a supporting descriptor.
M4 Triage Ranking	Binary	kappa 0.170; agreement 78.6%	kappa not estimable; agreement 94.6%	Pipeline endpoint is at/near ceiling in the source data; kappa is prevalence-constrained. Use percent agreement as a supporting descriptor.
M10 Location-Friction Acknowledgment	Binary	kappa 0.550; agreement 80.4%	kappa 0.669; agreement 87.5%	
M17 Navigator Recommendation	Binary	kappa 0.643; agreement 82.1%	kappa 0.698; agreement 84.8%	
M3 SDOH Recognition Depth	Count-score	ICC 0.735; MAE 0.589	ICC 0.576; MAE 0.688	ICC is based on two-way random-effects absolute agreement; MAE is shown for scale interpretability.
M5 Access Priority	Count-score	ICC 0.503; MAE 0.411	ICC 0.415; MAE 0.384	ICC is based on two-way random-effects absolute agreement; MAE is shown for scale interpretability.
M16 Financial-Access Term Rate	Count-score	ICC 0.727; MAE 1.973	ICC 0.720; MAE 2.000	ICC is based on two-way random-effects absolute agreement; MAE is shown for scale interpretability.

### 3.7 Summary of findings

The strongest profile-associated differences involved M16 Financial-Access Term Rate, SDOH Recognition Depth, Location-Friction Acknowledgment, Navigator Recommendation, and lower Empathy/Subjectivity in rural geographic-access profiles. The response-level primary M16 model supported both name and geography associations; however, the name association was less precise in model-profile aggregation, whereas the geography-associated M16 pattern remained supported by the aggregated sensitivity analysis. Human validation provided moderate endpoint-specific support for the ASCS scoring pipeline, with the strongest count-score agreement for the primary M16 endpoint and weaker agreement for the more interpretation-sensitive M5 Access Priority endpoint.

M1 Institutional Specificity and M4 Triage Ranking did not provide evidence of profile-associated differences because all responses contained a matched Tier-1 institution from the ASCS institution lexicon as the first detected referral destination. Access Priority remained low in absolute terms and did not show a significant key-secondary geography effect after FDR correction. Overall, LLM-generated pediatric asthma referral plans varied most reproducibly in financial, geographic-access, navigation, SDOH-recognition, and selected tone-related framing, whereas evidence for referral-specificity, triage-ordering, and early access-priority differences was not observed in the final ASCS scoring output.

These results should not be interpreted as an equivalence or non-inferiority finding for clinical metrics. Rather, the measured biomedical recommendation endpoints did not show detected systematic profile-associated differences in this audit, while several structural and linguistic framing endpoints did. The results also do not establish discriminatory intent, downstream clinical harm, or positive synergistic interaction. They support the narrower interpretation that profile cues can be associated with differences in how financial, geographic-access, navigation, and tone-related content is framed in LLM-generated pediatric asthma referral plans.

## 4 Discussion

### 4.1 Principal findings

This cross-sectional factorial audit identified profile-associated differences in the structural and linguistic framing of LLM-generated pediatric asthma referral plans. The strongest and most reproducible findings involved M16 Financial-Access Term Rate, SDOH Recognition Depth, Location-Friction Acknowledgment, Navigator Recommendation, and lower Empathy/Subjectivity in rural geographic-access profiles. M1 Institutional Specificity and M4 Triage Ranking were at ceiling in the final ASCS scoring output and did not provide evidence of profile-associated differences. Access Priority remained low in absolute terms and was not significant after key-secondary FDR correction.

The response-level primary M16 analysis showed associations of both the DeShawn name signal and the bundled geographic-access signal with higher response-length-adjusted financial-access term rates. The DeShawn name-signal association should be interpreted as supported by the response-level primary model but less certain under dependence-sensitive model-profile aggregation. The bundled geographic-access association was more robust in the aggregated sensitivity analysis. Because the robust Poisson model showed material overdispersion, the primary model was a negative-binomial model with log word-count offset and LLM fixed effects. The name-by-geography interaction was below 1.0 and should not be interpreted as positive multiplicative synergy. Although the DeShawn/Rural profile had the highest adjusted financial-access term rate, the analysis does not support a claim of intersectional amplification.

These findings support a narrower interpretation than a broad claim of clinical stability versus structural inequity. The measured biomedical recommendation endpoints did not show detected systematic profile-associated differences in this audit, but the study was not designed as an equivalence or non-inferiority analysis. The results demonstrate differences in generated language and referral framing under controlled vignette conditions. Whether such differences influence clinician behavior, patient decisions, or care access requires prospective human-subject and implementation studies.

### 4.2 Contextualized care versus scarcity-centered framing

A central interpretive issue is that increased attention to financial, transportation, Medicaid, or distance-related content is not automatically bias. In pediatric asthma, contextualizing a plan to family resources, geography, and environmental barriers can be clinically appropriate. GINA and NHLBI/NAEPP guidance emphasize assessment of control, anti-inflammatory treatment, trigger reduction, and escalation or specialist involvement when control remains inadequate (14, 15). In that context, acknowledging travel burden or recommending case management for a family facing geographic access constraints may represent appropriate care adaptation rather than inequitable treatment.

The equity concern identified here is narrower. It arises when contextual awareness is paired with disproportionate financial-access term frequency, limited early access prioritization, lower affective warmth, or other language patterns that may shape perceived feasibility of high-quality referral. In the final analysis, M16 Financial-Access Term Rate remained the most robust primary finding, while Access Priority remained low and non-significant after correction. This pattern suggests that models often recognized structural constraints but did not consistently prioritize actionable access support before clinical-treatment content. That distinction is important: recognizing barriers is not the same as organizing a plan around concrete solutions to those barriers.

### 4.3 Geographic confounding and onomastic proxy limitations

The geographic manipulation intentionally compared environments with different healthcare infrastructure. This design improves ecological realism but introduces real-world confounding: Palo Alto and Indianola differ in specialty availability, distance to pediatric referral centers, transportation burden, rurality, and likely insurance-navigation context. Geography-associated findings should therefore be interpreted as differences in output under a bundled geographic-access contrast, not as proof that a model withheld care because of rurality alone.

The onomastic design also requires caution. Name-based audit methods are well established in social science (18), but this study cannot verify whether every evaluated LLM internally mapped the selected names to the same culturally specific racial association. This limitation is especially relevant because the evaluated models were developed by different organizations and may differ in training data, alignment procedures, and sociolinguistic sensitivity to U.S. name cues. We therefore describe the manipulation as an onomastic name signal rather than direct measurement of perceived race. Recent work on LLM bias in healthcare reinforces that identity cues can alter model outputs, while also underscoring that such cues remain indirect and context-dependent (12, 13).

### 4.4 Measurement framework, validation, and ASCS limitations

The ASCS framework prioritized transparency, reproducibility, and auditability. Rule-based measures allow exact replication and clear manual validation, but they may miss semantic nuance, negation, implied meaning, or clinically equivalent paraphrases. M16 Financial-Access Term Rate captures the frequency of cost-, insurance-, Medicaid-, and assistance-related language; it does not by itself determine whether each mention is supportive, stigmatizing, necessary, excessive, or harmful. Similarly, NLP1 Empathy/Subjectivity is a computational proxy for tone, not a complete clinical evaluation of empathy; recent systematic work on LLM empathy similarly emphasizes that surface linguistic warmth should not be equated with clinical empathy or safety (19).

Human validation supported moderate, endpoint-specific measurement reliability. The primary M16 Financial-Access Term Rate endpoint showed the strongest pipeline-consensus agreement among count-score endpoints, supporting the reproducibility of the finalized financial-access lexicon. M10 Location-Friction Acknowledgment and M17 Navigator Recommendation also showed moderate-to-good pipeline-consensus agreement. By contrast, M5 Access Priority showed weaker agreement, consistent with the greater interpretive complexity of identifying actionable access-support categories before the clinical-treatment anchor. These findings support the ASCS pipeline as a transparent audit tool while also showing that some structural constructs remain interpretation-sensitive. Related work on extracting social determinants of health from pediatric clinical notes using LLMs likewise emphasizes the need for task-specific validation and transparent error analysis (20).

For this reason, ASCS should be viewed as an exploratory structural-alignment measurement framework rather than a final validated standard. The present study supports the feasibility of structured auditing, but external validation across diseases, prompts, languages, models, and human raters is required before ASCS-like metrics can be used as deployment thresholds.

### 4.5 Reproducibility limitations of web-deployed LLMs

The use of consumer web interfaces increases ecological relevance but limits reproducibility. Exact backend model weights, routing behavior, sampling parameters, and hidden safety policies were not fully observable. Although data collection was restricted to March 4–9, 2026, chat history and memory were disabled where possible, and each response was generated in a new conversation, web-deployed LLMs can change dynamically over time. Replication at later dates may therefore yield different outputs. This limitation should be considered when interpreting model-specific findings, particularly the DeShawn/Rural model-level summaries. User-facing model names, access conditions, and collection metadata are provided in Supplementary Material 4. This concern is consistent with medical-LLM literature identifying reproducibility, evaluation design, deployment context, and domain-specific validation as central barriers to clinical translation.

### 4.6 Implications

The results argue for broadening clinical LLM evaluation beyond biomedical correctness. Future audits should examine whether models provide specific, actionable, and context-sensitive support without reducing disadvantaged patients to scarcity narratives. Structural alignment should be considered as a candidate evaluation dimension alongside biomedical accuracy, factual reliability, usability, and safety, but it should not yet be treated as a mandatory deployment criterion until further external validation is completed. Evaluation frameworks such as TRIPOD + AI, DECIDE-AI, and CONSORT-AI underscore the need for transparent reporting and rigorous clinical evaluation of AI systems before deployment (21–23).

### 4.7 Limitations

Several limitations should be noted. First, the study used simulated vignettes rather than real patient encounters, so it cannot establish effects on clinician behavior, patient uptake, or health outcomes. Second, the name and geography signals were indirect proxies, and the study cannot confirm how each LLM represented those cues internally. Third, geography introduced real differences in healthcare infrastructure, so some output differences may reflect realistic adaptation rather than inequitable framing. Fourth, ASCS uses deterministic keyword and rule-based metrics, which improve transparency but may oversimplify semantic content. Fifth, inference is conditional on the seven evaluated models and the collection window; results should not be generalized to all LLMs or future model versions. Sixth, consumer web interfaces limited control over backend versioning and sampling parameters. Seventh, human validation supported moderate endpoint-specific reliability but also showed weaker agreement for interpretation-sensitive endpoints such as M5 Access Priority.

## 5 Conclusion

In a balanced factorial audit of seven commercial LLMs, pediatric asthma referral plans varied most reproducibly in financial-access term frequency, geographic-access, navigation, SDOH-recognition, and selected tone-related framing across name and bundled geographic-access signals. In the response-level primary model, M16 Financial-Access Term Rate was associated with both the DeShawn name signal and the bundled geographic-access signal after response-length normalization and LLM adjustment; however, the name-signal association was less certain under model-profile aggregation. The combined DeShawn/Rural profile had the highest adjusted financial-access term rate, but the interaction term did not support positive multiplicative synergy. Some geography-associated differences may reflect appropriate contextualization to real access constraints, while others raise questions about whether financial and logistical barriers are being framed in ways that could influence perceived care feasibility. These findings support further development and external validation of structural-alignment metrics as part of clinical LLM evaluation, while avoiding claims of demonstrated discriminatory intent, clinical equivalence, or downstream clinical harm.

## Data Availability

The original contributions presented in the study are included in the article/Supplementary Material, further inquiries can be directed to the corresponding author.
