# Supplementary material for: Profile-associated financial and access-related framing in LLM-generated pediatric asthma referral plans: a factorial audit of seven large language models
Source: Front Digit Health. 2026 Jul 7;8:1825576. doi: 10.3389/fdgth.2026.1825576 (PMC13385167; doi:10.3389/fdgth.2026.1825576)
Supplement: Supplementary file 2 [file Supplementaryfile2.docx]

**Supplementary Materials 2**

**Supplementary 2 Table S1. Profile-level descriptive statistics for key ASCS metrics.**

| **Metric** | **Liam / Urban** | **DeShawn / Urban** | **Liam / Rural** | **DeShawn / Rural** |
| --- | --- | --- | --- | --- |
| M1_Inst_Specificity | 100.0% (140/140) | 100.0% (140/140) | 100.0% (140/140) | 100.0% (140/140) |
| M3_SDOH_Depth | 3.800 (0.883) | 4.000 (0.865) | 4.714 (0.527) | 4.729 (0.492) |
| M4_Triage_Ranking | 100.0% (140/140) | 100.0% (140/140) | 100.0% (140/140) | 100.0% (140/140) |
| M5_Access_Priority | 0.150 (0.358) | 0.164 (0.409) | 0.207 (0.543) | 0.229 (0.500) |
| M10_Location_Friction | 67.9% (95/140) | 67.1% (94/140) | 99.3% (139/140) | 100.0% (140/140) |
| M16_Financial_Access_Term_Count | 1.936 (2.047) | 2.843 (2.634) | 4.743 (3.637) | 5.736 (4.421) |
| M16_rate1000 | 2.002 (2.038) | 2.942 (2.596) | 4.732 (2.930) | 5.539 (3.574) |
| M17_Navigator_Rec | 27.9% (39/140) | 45.7% (64/140) | 70.7% (99/140) | 77.9% (109/140) |
| NLP1_Empathy_Subjectivity | 0.376 (0.047) | 0.369 (0.047) | 0.362 (0.043) | 0.357 (0.045) |
| NLP2_Polarity | 0.082 (0.037) | 0.083 (0.034) | 0.074 (0.031) | 0.066 (0.030) |
| Q9_Word_Count | 907.279 (238.233) | 905.071 (256.357) | 936.650 (243.896) | 954.586 (284.994) |

**Supplementary 2 Table S2. Count-model diagnostics.**

| **Outcome** | **Offset** | **Pearson_dispersion** | **Material_overdispersion** |
| --- | --- | --- | --- |
| M16_Financial_Access_Term_Count | log word count | 1.5626 | Yes |
| M3_SDOH_Depth | None | 0.113 | No |
| M5_Access_Priority | None | 1.0082 | No |

**Supplementary 2 Table S3. Negative-binomial analyses for selected count endpoints.**

| **Outcome** | **Effect** | **Estimate_type** | **Estimate** | **CI_low** | **CI_high** | **p** | **alpha** | **Converged** |
| --- | --- | --- | --- | --- | --- | --- | --- | --- |
| M16_Financial_Access_Term_Count | Name signal: DeShawn vs Liam | IRR | 1.47328 | 1.22657 | 1.76963 | 3e-05 | 0.14811 | True |
| M16_Financial_Access_Term_Count | Geography signal: Rural vs Urban | IRR | 2.4011 | 2.02125 | 2.85234 | 0.0 | 0.14811 | True |
| M16_Financial_Access_Term_Count | Name x Geography interaction | IRR | 0.79147 | 0.62787 | 0.99768 | 0.04774 | 0.14811 | True |
| M5_Access_Priority | Name signal: DeShawn vs Liam | RR | 1.09394 | 0.6016 | 1.98921 | 0.76851 | 0.0973 | True |
| M5_Access_Priority | Geography signal: Rural vs Urban | RR | 1.38408 | 0.78394 | 2.44366 | 0.26242 | 0.0973 | True |
| M5_Access_Priority | Name x Geography interaction | RR | 1.0097 | 0.4601 | 2.21583 | 0.9808 | 0.0973 | True |

**Supplementary 2 Table S4. Robust linear sensitivity analyses for M3 and M5.**

| **Outcome_label** | **Effect** | **Estimate_type** | **Estimate** | **CI_low** | **CI_high** | **p** | **q_BH_bounded** |
| --- | --- | --- | --- | --- | --- | --- | --- |
| M3_SDOH_Depth | Name signal: DeShawn vs Liam | AMD | 0.2 | 0.00944 | 0.39056 | 0.03967 | 0.11902 |
| M3_SDOH_Depth | Geography signal: Rural vs Urban | AMD | 0.91429 | 0.75047 | 1.0781 | 0.0 | 0.0 |
| M3_SDOH_Depth | Name x Geography interaction | AMD | -0.18571 | -0.40901 | 0.03759 | 0.10308 | 0.20616 |
| M5_Access_Priority | Name signal: DeShawn vs Liam | AMD | 0.01429 | -0.0715 | 0.10008 | 0.74414 | 0.89297 |
| M5_Access_Priority | Geography signal: Rural vs Urban | AMD | 0.05714 | -0.04854 | 0.16283 | 0.28925 | 0.43388 |
| M5_Access_Priority | Name x Geography interaction | AMD | 0.00714 | -0.1412 | 0.15549 | 0.92481 | 0.92481 |

**Supplementary 2 Table S5. Leave-one-LLM-out sensitivity analysis.**

| **Excluded_LLM** | **Effect** | **IRR** | **CI_low** | **CI_high** | **p** |
| --- | --- | --- | --- | --- | --- |
| ChatGPT | Name signal | 1.4987 | 1.23512 | 1.81852 | 4e-05 |
| ChatGPT | Geography signal | 2.40753 | 2.00594 | 2.88952 | 0.0 |
| ChatGPT | Interaction | 0.77824 | 0.60884 | 0.99479 | 0.04532 |
| Claude | Name signal | 1.48991 | 1.21408 | 1.82839 | 0.00013 |
| Claude | Geography signal | 2.21377 | 1.82126 | 2.69088 | 0.0 |
| Claude | Interaction | 0.82089 | 0.63208 | 1.0661 | 0.13887 |
| DeepSeek | Name signal | 1.4515 | 1.19113 | 1.76879 | 0.00022 |
| DeepSeek | Geography signal | 2.36775 | 1.9652 | 2.85276 | 0.0 |
| DeepSeek | Interaction | 0.77955 | 0.60634 | 1.00223 | 0.05206 |
| GLM | Name signal | 1.49579 | 1.22223 | 1.83058 | 9e-05 |
| GLM | Geography signal | 2.77347 | 2.30249 | 3.34079 | 0.0 |
| GLM | Interaction | 0.75539 | 0.5889 | 0.96895 | 0.02722 |
| Gemini | Name signal | 1.389 | 1.15362 | 1.67241 | 0.00052 |
| Gemini | Geography signal | 2.21574 | 1.86156 | 2.6373 | 0.0 |
| Gemini | Interaction | 0.87007 | 0.68794 | 1.10042 | 0.24546 |
| Grok | Name signal | 1.53658 | 1.26714 | 1.8633 | 1e-05 |
| Grok | Geography signal | 2.47027 | 2.05951 | 2.96296 | 0.0 |
| Grok | Interaction | 0.76937 | 0.603 | 0.98164 | 0.03494 |
| Qwen | Name signal | 1.4616 | 1.18296 | 1.80587 | 0.00044 |
| Qwen | Geography signal | 2.42212 | 1.98731 | 2.95207 | 0.0 |
| Qwen | Interaction | 0.76857 | 0.58843 | 1.00387 | 0.0534 |

**Supplementary 2 Table S6. Model-profile cell-level aggregated sensitivity analyses.**

| **Outcome_label** | **Effect** | **Estimate_type** | **Estimate** | **CI_low** | **CI_high** | **p** | **q_BH_aggregate** |
| --- | --- | --- | --- | --- | --- | --- | --- |
| M16 rate/1000 | Name signal: DeShawn vs Liam | AMD | 0.94008 | -0.08128 | 1.96143 | 0.07123 | 0.24383 |
| M16 rate/1000 | Geography signal: Rural vs Urban | AMD | 2.72955 | 1.57405 | 3.88505 | 0.0 | 2e-05 |
| M16 rate/1000 | Name x Geography interaction | AMD | -0.13312 | -1.74134 | 1.4751 | 0.87112 | 0.95301 |
| M3 mean | Name signal: DeShawn vs Liam | AMD | 0.2 | -0.03466 | 0.43466 | 0.09482 | 0.24383 |
| M3 mean | Geography signal: Rural vs Urban | AMD | 0.91429 | 0.67633 | 1.15225 | 0.0 | 0.0 |
| M3 mean | Name x Geography interaction | AMD | -0.18571 | -0.50424 | 0.13281 | 0.25314 | 0.50628 |
| M5 mean | Name signal: DeShawn vs Liam | AMD | 0.01429 | -0.12757 | 0.15614 | 0.84353 | 0.95301 |
| M5 mean | Geography signal: Rural vs Urban | AMD | 0.05714 | -0.11344 | 0.22773 | 0.51147 | 0.81379 |
| M5 mean | Name x Geography interaction | AMD | 0.00714 | -0.23042 | 0.24471 | 0.95301 | 0.95301 |
| M10 proportion | Name signal: DeShawn vs Liam | AMD | -0.00714 | -0.1557 | 0.14142 | 0.92492 | 0.95301 |
| M10 proportion | Geography signal: Rural vs Urban | AMD | 0.31429 | 0.20992 | 0.41865 | 0.0 | 0.0 |
| M10 proportion | Name x Geography interaction | AMD | 0.01429 | -0.15867 | 0.18724 | 0.87139 | 0.95301 |
| M17 proportion | Name signal: DeShawn vs Liam | AMD | 0.17857 | -0.09024 | 0.44738 | 0.1929 | 0.43403 |
| M17 proportion | Geography signal: Rural vs Urban | AMD | 0.42857 | 0.16873 | 0.68841 | 0.00123 | 0.00552 |
| M17 proportion | Name x Geography interaction | AMD | -0.10714 | -0.45197 | 0.23769 | 0.54253 | 0.81379 |
| NLP1 mean | Name signal: DeShawn vs Liam | AMD | -0.00669 | -0.02238 | 0.009 | 0.40314 | 0.72565 |
| NLP1 mean | Geography signal: Rural vs Urban | AMD | -0.01347 | -0.0288 | 0.00185 | 0.08486 | 0.24383 |
| NLP1 mean | Name x Geography interaction | AMD | 0.00098 | -0.02135 | 0.0233 | 0.93155 | 0.95301 |

**Supplementary 2 Table S7. Model-level heterogeneity tests.**

| **Outcome** | **Kruskal_Wallis_H** | **p** | **q_BH_model_heterogeneity** |
| --- | --- | --- | --- |
| M16_Financial_Access_Term_Count | 69.14885 | 0.0 | 0.0 |
| M16_rate1000 | 37.94563 | 0.0 | 0.0 |
| Q9_Word_Count | 112.58962 | 0.0 | 0.0 |
| M3_SDOH_Depth | 19.186 | 0.00386 | 0.00386 |
| M5_Access_Priority | 20.2139 | 0.00254 | 0.0029 |
| M10_Location_Friction | Not estimable | Not estimable | No between-model variation; all DeShawn/Rural responses were positive |
| M17_Navigator_Rec | 49.44599 | 0.0 | 0.0 |
| NLP1_Empathy_Subjectivity | 33.59351 | 1e-05 | 1e-05 |

**Supplementary 2 Table S8. M5 category distribution.**

| **Category** | **Count** | **Percent** |
| --- | --- | --- |
| none | 467 | 83.4 |
| navigation_or_case_management | 73 | 13.0 |
| home_school_community_support | 7 | 1.2 |
| scheduling_referral_coordination | 4 | 0.7 |
| navigation_or_case_management;home_school_community_support | 4 | 0.7 |
| navigation_or_case_management;scheduling_referral_coordination | 2 | 0.4 |
| transportation_assistance;insurance_financial_navigation;navigation_or_case_management | 1 | 0.2 |
| transportation_assistance;insurance_financial_navigation;navigation_or_case_management;scheduling_referral_coordination | 1 | 0.2 |
| transportation_assistance;home_school_community_support | 1 | 0.2 |
