# Supplementary material for: Profile-associated financial and access-related framing in LLM-generated pediatric asthma referral plans: a factorial audit of seven large language models
Source: Front Digit Health. 2026 Jul 7;8:1825576. doi: 10.3389/fdgth.2026.1825576 (PMC13385167; doi:10.3389/fdgth.2026.1825576)
Supplement: Supplementary file 4 [file Supplementaryfile4.docx]

**Supplementary Materials 4**

*Model access metadata and data-collection audit trail*

This supplementary file documents the user-facing model names, access conditions, collection window, and interface-level observability for the final analytic corpus. Because all systems were accessed through consumer web interfaces, exact backend model weights, dynamic routing behavior, hidden system prompts, and sampling parameters were not observable. The information below should therefore be interpreted as a structured audit trail of the user-facing collection conditions rather than a verification of hidden backend configurations.

# **S13. User-facing model identity and access conditions**

| **Model label** | **Provider** | **User-facing model name** | **Access mode** | **Account/session status** | **Collection window** | **Outputs** |
| --- | --- | --- | --- | --- | --- | --- |
| ChatGPT | OpenAI | GPT-5.3 instant | Standard web interface | Logged-in web session | March 4-9, 2026 | 80 |
| Claude | Anthropic | Claude 4.6 Sonnet | Standard web interface | Logged-in web session | March 4-9, 2026 | 80 |
| DeepSeek | DeepSeek AI | DeepSeek-V3.2 | Standard web interface | Logged-in web session | March 4-9, 2026 | 80 |
| GLM | Zhipu AI | GLM-5 | Standard web interface | Logged-in web session | March 4-9, 2026 | 80 |
| Gemini | Google | Gemini 3 thinking | Standard web interface | Logged-in web session | March 4-9, 2026 | 80 |
| Grok | xAI | Grok-4.1 Auto | Standard web interface | Logged-in web session | March 4-9, 2026 | 80 |
| Qwen | Alibaba | Qwen 3.5 Plus Auto | Standard web interface | Logged-in web session | March 4-9, 2026 | 80 |

*Note: User-facing model names are reported as displayed or selected by the collectors during the collection window. Providers may update routing, backend models, or safety layers over time without changing the visible interface.*

# **S14. Interface controls, observability, and response-generation protocol**

| **Model** | **Memory / chat-history setting** | **Web / search setting** | **Temperature / sampling** | **System prompt** | **Backend version observability** | **New conversation per response** | **Prompt source** |
| --- | --- | --- | --- | --- | --- | --- | --- |
| ChatGPT | Disabled where available; otherwise not user-controllable or not visible | Default initial setting of the interface | Not visible or user-adjustable | Not visible | Exact backend weights/routing not visible | Yes | Locked prompt template in Supplementary Materials 1 |
| Claude | Disabled where available; otherwise not user-controllable or not visible | Default initial setting of the interface | Not visible or user-adjustable | Not visible | Exact backend weights/routing not visible | Yes | Locked prompt template in Supplementary Materials 1 |
| DeepSeek | Disabled where available; otherwise not user-controllable or not visible | Default initial setting of the interface | Not visible or user-adjustable | Not visible | Exact backend weights/routing not visible | Yes | Locked prompt template in Supplementary Materials 1 |
| GLM | Disabled where available; otherwise not user-controllable or not visible | Default initial setting of the interface | Not visible or user-adjustable | Not visible | Exact backend weights/routing not visible | Yes | Locked prompt template in Supplementary Materials 1 |
| Gemini | Disabled where available; otherwise not user-controllable or not visible | Default initial setting of the interface | Not visible or user-adjustable | Not visible | Exact backend weights/routing not visible | Yes | Locked prompt template in Supplementary Materials 1 |
| Grok | Disabled where available; otherwise not user-controllable or not visible | Default initial setting of the interface | Not visible or user-adjustable | Not visible | Exact backend weights/routing not visible | Yes | Locked prompt template in Supplementary Materials 1 |
| Qwen | Disabled where available; otherwise not user-controllable or not visible | Default initial setting of the interface | Not visible or user-adjustable | Not visible | Exact backend weights/routing not visible | Yes | Locked prompt template in Supplementary Materials 1 |

# **S15. Data-collection design summary**

| **Item** | **Recorded value** |
| --- | --- |
| Final analytic corpus | 560 complete LLM-generated pediatric asthma referral plans |
| Factorial design | 2 x 2 design crossing patient name signal and bundled geographic-access signal |
| Profiles | Liam/Urban, DeShawn/Urban, Liam/Rural, DeShawn/Rural |
| Clinical scenario | 5-year-old boy with moderate persistent asthma, persistent nocturnal symptoms despite low-dose ICS, FEV1 70% predicted, ACT score 16 |
| Manipulated fields | Patient name and patient address only |
| Responses per model-profile cell | 20 separately initiated response generations |
| Responses per model | 80 |
| Responses per profile | 140 |
| Total evaluated models | 7 |
| Collection window | March 4-9, 2026 |
| Response capture | Complete free-text referral plans were recorded and scored at response level |
| Primary scoring source | ASCS scoring pipeline and locked prompt documentation in Supplementary Materials 1 |

# **S16. Collection-log structure used for audit-trail verification**

The response-level collection log and raw-response table should retain, at minimum, the fields below. These fields allow independent verification that the final corpus is balanced across models, profiles, and iterations and that each response can be linked to the prompt version and scoring output.

| **Collection-log field** | **Description** |
| --- | --- |
| Response_ID | Unique response identifier linking raw text, ASCS scores, and analysis dataset |
| Model_label | ChatGPT, Claude, DeepSeek, GLM, Gemini, Grok, or Qwen |
| Provider | Model provider/developer |
| User_facing_model_name | Displayed or selected model name during the collection window |
| Profile | Liam/Urban, DeShawn/Urban, Liam/Rural, or DeShawn/Rural |
| Iteration | 1-20 within each model-profile cell |
| Collection_date | Calendar date of response collection within March 4-9, 2026 |
| Prompt_version | Locked prompt template; exact text in Supplementary Materials 1 |
| New_conversation | Yes for every response |
| Raw_response_text | Complete unedited model output used for ASCS scoring |
| Collector_initials | Initials or identifier of the person collecting the output, if available |
| Notes | Platform interruption, regeneration, visible retrieval indicator, or other collection notes, if applicable |

# **S17. Reproducibility and observability checklist**

| **Audit-trail element** | **Location or status** |
| --- | --- |
| Locked prompt template | Provided in Supplementary Materials 1 |
| Four instantiated prompts | Provided in Supplementary Materials 1 |
| User-facing model names | Provided in Supplementary Table S13 |
| Collection window | March 4-9, 2026, reported in Methods and Supplementary Table S13 |
| Response-level balance | Seven models x four profiles x 20 responses per cell = 560 responses |
| New conversation per response | Recorded as part of the collection protocol |
| Memory/chat-history control | Disabled where available; otherwise not user-controllable or not visible |
| Web/search setting | Default initial setting of each interface |
| Sampling parameters | Not visible or user-adjustable in the consumer web interfaces |
| Backend weights/routing | Not observable; inference is conditional on user-facing deployed systems during the collection window |
| Raw response linkage | Response_ID links raw text, scoring output, and analysis dataset |
| Scoring code | Provided in Supplementary Materials 1 |
| Human validation codebook | Provided in Supplementary Materials 3 |

This audit-trail documentation is intended to improve transparency for a study using dynamic consumer web interfaces. It does not remove the reproducibility limitations inherent to web-deployed LLMs. Replication at later dates may produce different outputs if providers update user-facing models, routing policies, retrieval behavior, or safety layers.
